# AHP-Based Evaluation of Hybrid Kenaf/Flax/Glass Fiber-Reinforced Biocomposites for Unmanned Maritime Vehicle Applications

**DOI:** 10.3390/ma18163731

**Published:** 2025-08-08

**Authors:** Yang Huang, Mohamed Thariq Hameed Sultan, Andrzej Łukaszewicz, Farah Syazwani Shahar, Zbigniew Oksiuta

**Affiliations:** 1 Department of Aerospace Engineering, Faculty of Engineering, Universiti Putra Malaysia, Serdang 43400, Selangor, Malaysia; huangyang821210@hotmail.com (Y.H.); farahsyazwani@upm.edu.my (F.S.S.); 2 College of Civil Engineering and Architecture, Beibu Gulf University, Qinzhou 535011, China; 3 Laboratory of Biocomposite Technology, Institute of Tropical Forestry and Forest Products (INTROP), Universiti Putra Malaysia, Serdang 43400, Selangor, Malaysia; 4 Aerospace Malaysia Innovation Centre (944751-A), Prime Minister’s Department, MIGHT Partnership Hub, Jalan Impact, Cyberjaya 63000, Selangor, Malaysia; 5 Institute of Mechanical Engineering, Faculty of Mechanical Engineering, Bialystok University of Technology, 45C Wiejska St., 15-351 Bialystok, Poland; 6 Institute of Biomedical Engineering, Faculty of Mechanical Engineering, Bialystok University of Technology, 45C Wiejska St., 15-351 Bialystok, Poland; z.oksiuta@pb.edu.pl

**Keywords:** unmanned maritime vehicle, kenaf fiber, glass fiber, flax fiber, tensile properties, flexural properties, seawater aging, fiber-reinforced composite, analytic hierarchy process (AHP), marine application

## Abstract

Unmanned maritime vehicles (UMVs) have become essential tools in marine research and monitoring, significantly enhancing operational efficiency and reducing risks and costs. Fiber-reinforced composites have been widely used in marine applications due to their excellent characteristics. However, environmental concerns and the pursuit of sustainable development goals have driven the development of environmentally friendly materials. The development of eco-friendly biocomposites for UMV construction can effectively reduce the environmental impact of marine equipment. This study investigates the effects of seawater aging on kenaf/flax/glass-fiber-reinforced composites under artificial seawater conditions and determines their ranking for UMVs using the Analytic Hierarchy Process (AHP). These hybrid composites, fabricated with various stacking sequences, were prepared using a combination of hand lay-up and vacuum bagging techniques. All plant fibers underwent sodium hydroxide treatment to eliminate impurities and enhance interfacial bonding, while nano-silica was incorporated into the epoxy matrix to improve overall performance. After 50 days of immersion in artificial seawater, mechanical tests were conducted to evaluate the extent of changes in mechanical properties. Subsequently, the AHP analysis was performed based on three main criteria and thirteen sub-criteria to determine the most suitable configuration for marine applications. The results demonstrate that the stacking sequence plays a critical role in resisting seawater-induced degradation and maintaining mechanical performance. GKFKG exhibited the highest retention rates for both tensile strength (86.77%) and flexural strength (88.36%). Furthermore, the global priority vector derived from the AHP analysis indicates that hybrid composites consisting of kenaf, flax, and glass fibers consistently ranked highest. The optimum configuration among these hybrid composites was determined to be GKFKG, followed by GFKFG, GKKKG, and GKGKG.

## 1. Introduction

In recent years, the rapid evolution of intelligent control systems has greatly facilitated the widespread application of unmanned and autonomous technologies in various fields, including industrial automation, terrestrial transportation, aerospace, marine operations, and military applications [1]. Unmanned systems have notable advantages in terms of reliable remote controllability, improved task efficiency, cost efficiency, extended endurance, and reduced operational risk [2]. In the marine environment, unmanned maritime vehicles (UMVs) represent essential equipment and can be categorized into unmanned surface vehicles (USVs) and unmanned underwater vehicles (UUVs) based on their respective operational domains [3]. They can be integrated with unmanned aerial vehicles (UAVs) and other technological platforms to establish a collaborative operational system [4]. Therefore, these unmanned platforms are expected to enhance mission success rates, attracting considerable attention from both academic and industrial areas [5]. These UMVs have expanded their application domains beyond traditional military operations and scientific exploration, spanning various civilian and commercial sectors [6]. Therefore, selecting an appropriate material plays a critical role in the design and fabrication of UMVs, as it directly affects mechanical reliability, resistance to harsh marine environments, and operational adaptability. Generally, lightweight construction, high mechanical strength, and resistance to seawater-induced aging are key performance requirements. Compared with isotropic materials, composite materials, particularly fiber-reinforced composites, can demonstrate superior strength-to-weight ratios [7]. Furthermore, these composites can be engineered and tailored through design optimization to produce strong yet thinner structural walls, thereby reducing the overall structural mass and increasing the safety level [7].

Fiber-reinforced composites (FRCs) are widely utilized in this field due to their advantageous combination of lightweight characteristics and high mechanical strength [8]. The traditional reinforcement materials are mainly synthetic fibers. Although synthetic fibers have excellent mechanical properties and stability in composite reinforcement, their manufacturing and recycling processes pose challenges to the environment [9]. This reason has prompted the development of natural-fiber-reinforced composites, as natural fibers are renewable and biodegradable [8]. In addition, plant fibers, with low density and a low price, are considered an important alternative to synthetic fibers in fabricating FRCs. Furthermore, these cellulose fibers are much safer, reducing the risk of allergic reactions in exposed individuals during manufacture and utilization [10]. Although plant fibers are regarded as viable alternatives to synthetic fibers, composites reinforced with a single type of plant fiber often face limitations in practical applications. Therefore, FRCs made from plant fibers have not yet fully replaced their synthetic counterparts due to limitations in overall performance, stability, and environmental adaptability. Hybridization represents an effective strategy to overcome the limitations associated with single-fiber-reinforced composites [11]. This approach allows FRCs to satisfy the diverse requirements associated with various application scenarios. The mechanical properties of FRCs can be significantly enhanced by employing fiber hybridization techniques and optimizing stacking sequence [12]. In addition, the strategy can effectively improve the resistance of FRCs to water-induced degradation [13]. Previous research has demonstrated that kenaf fiber composites exhibit inferior tensile and flexural properties compared to their glass fiber counterparts, and the hybrid configuration has a stronger resistance capability compared to the pure kenaf fiber composite material [14]. In hybrid kenaf–glass fiber composites, both tensile and flexural strengths increase proportionally with a higher glass fiber content [15]. Additionally, optimizing the stacking sequence of natural fiber layers can enhance the mechanical performance of hybrid composites [16]. Generally, hybrid composites exhibit water absorption characteristics intermediate between that of composites made from individual fiber types [17]. Moreover, the combination of excellent product design and matching manufacturing techniques can enhance the quality of materials [18]. Furthermore, the development of high-performance biocomposites satisfies the sustainability demands under the framework of the United Nations Sustainable Development Goals [19].

Aquatic environments are not conducive for FRCs. Water molecules entering FRCs can trigger material swelling, debonding at the fiber–matrix interface, microcrack propagation, and fiber bundle splitting [20]. Generally, moisture ingress in composite materials exists primarily as combined water and free water [21]. In addition, the water ingress causes changes in the density and surface roughness of the FRCs [20]. Moreover, water absorption leads to the deterioration of both the mechanical properties of the reinforcing fibers and the matrix in FRCs [22]. In such conditions, the hygroscopicity of plant fibers also leads to adverse effects. Nosbi et al. [23] reported that kenaf fibers have many hydroxyl groups on their surface. When exposed to humid environments, these hydroxyl groups can easily form hydrogen bonds between the fiber and polymer. The increase in hydrogen bonding will lead to a reduction in the mechanical properties of composite materials. Then, these reasons can impact the overall performance of composites in aquatic conditions. Prolonged aquatic exposure can lead to aggravated mechanical deterioration, attributed to resin swelling, resin plasticization, and fiber/matrix interface debonding [21,24]. The water absorption behavior of composite materials is influenced by multiple factors. These may result from the material’s intrinsic characteristics (such as the type of reinforcement fiber, matrix composition, and fabrication process) or from external environmental variables (including the immersion medium, temperature, and exposure duration). For example, Yan and Chouw [25] evaluated the long-term durability of flax fabric reinforced epoxy composites under water, seawater, and 5% NaOH conditions. After one year of aging, the composites exhibited significant moisture uptake and mechanical degradation. The composites exhibited a reduction of 22.6–31.1% in tensile strength, 24.0–36.4% in tensile modulus, 9.3–23.5% in flexural strength, and 13.9–25.2% in flexural modulus. The effects of 5% NaOH and seawater caused much more deterioration than those of freshwater.

Due to its high salinity and corrosive characteristics, seawater significantly undermines the structural integrity and durability of marine engineering structures [26]. For example, the hydrolytic degradation of cellulose is accelerated in seawater due to the complex ionic composition of seawater [23]. Therefore, the ions in seawater can cause significant damage to natural-fiber-reinforced composites (NFRCs) [27]. Researchers have been attempting to demonstrate the potential of NFRCs for marine applications [28,29,30,31]. Davies et al. [31] observed that immersing flax/PLA composites in seawater at 40 °C for up to nine months led to a tensile strength reduction of 60%, accompanied by a weight increase of 10%. Ambekar et al. [32] made PALF/epoxy composites and soaked them in 40 °C artificial seawater for 70 days. They found that the tensile strength and modulus of the epoxy PALF composites were reduced by 23% and 15% after seawater aging. In contrast, for the group with 1.5 wt.% nanoclay, tensile strength and modulus decreased by 16% and 11%, respectively. The ions in seawater can accumulate in surface and internal voids, forming barriers that impede water diffusion and reduce the overall moisture absorption [20,23]. This indicates that the moisture uptake of the composite material in seawater is lower than that in freshwater, and the diffusion rate of seawater within the composites is reduced compared to that of freshwater [33]. Nevertheless, reducing the moisture absorption of composite materials is a key issue in marine environment applications requiring resolution. NFRCs made with large-diameter fibers have lower water absorption than those made with small-diameter fibers due to the reduced contact area between the reinforcing fibers and the matrix [34]. Moreover, hybridization with glass fibers plays a positive role in improving the moisture resistance of composite materials in humid environments [35]. Mishra et al. [36] reported that hybridization of kenaf fibers with glass fibers effectively inhibited the penetration of water molecules, particularly when the glass fibers were placed on the outer surfaces of the composite.

Alkali treatment, a surface modification technique, is an effective method to improve the performance of NFRCs [37,38,39]. This method eliminates non-cellulosic components, such as hemicellulose, lignin, and waxes, while also removing surface impurities [10]. Moreover, the treatment alters the surface morphology of the fibers, thereby enhancing their mechanical performance and improving the interfacial adhesion between the fibers and the polymer matrix [8]. Verma et al. [12] reported that treatment with a 5% *w*/*v* concentration of NaOH solution led to respective increases of 81%, 114%, and 42% in the tensile strength, tensile modulus, and elongation at break of kenaf fibers. However, a high-concentration NaOH solution may exert deleterious effects on the fibrous texture, particularly under prolonged exposure conditions. Ismail [40] treated kenaf fibers with 6 wt.% NaOH solution for 24 h and found that kenaf fibers became finer and brittle. Another effective strategy for enhancing the performance of composite materials involves the incorporation of nanoparticles. The capacity of NFRC to resist moisture can be enhanced through the incorporation of nanofillers, which can fill pores and gaps in the interfacial region and improve interfacial adhesion to reduce moisture ingress [10]. Among nanofillers, silica has been proven to improve the performance and durability of composite materials [41,42]; however, excessive concentrations of nano-silica may result in regional agglomeration, leading to an opposite outcome. According to Sagari and Prasad [43], the incorporation of nano-silica into jute/kenaf-fiber-reinforced composites effectively reduces void formation and moisture absorption but also enhances mechanical performance. The performance of the hybrid composites progressively improved with increasing nano-silica loading, reaching an optimal value at a concentration of 4.5 wt.%. Moreover, Emrahi et al. [44] found that incorporating 1–3 wt.% nano-silica into carbon-fiber-reinforced polymer (CFRP) improved the tensile, flexural, and compressive strengths of the samples. However, concentrations exceeding 3% resulted in a decline in these properties.

The purpose of multi-criteria decision-making (MCDM) is to identify the most suitable solution from a set of alternatives by applying rational and systematic methods [45]. The Analytic Hierarchy Process (AHP), developed by Thomas L. Saaty in the 1970s, is a mathematical solution designed to support the organization and analysis of complex decision-making problems [46,47]. This MCDM tool is particularly effective in addressing problems involving intricate interrelationships among factors [48]. Therefore, the AHP can enable a comprehensive evaluation of differences in material properties and performance to identify the most optimal selection. For example, Naveen et al. [49] employed the AHP to identify the most suitable natural fiber for hybridization with Kevlar 29 in the design of polymer composites for personal body armor. They established a three-level hierarchical framework that incorporates fourteen alternative natural fibers and seven evaluation criteria. Following the analysis, the Cocos nucifera sheath obtained the highest priority vector, identifying it as the most promising candidate among the evaluated natural fibers. Similarly, Balakrishnan et al. [50] applied the AHP method to assess thirteen natural fiber alternatives for suitability in fabricating pultruded composites. They further highlighted that AHP serves as an effective multi-criteria decision-making tool, enabling researchers and engineers to systematically carry out selection and problem-solving tasks across various applications. This study fabricates four hybrid kenaf/flax/glass-fiber-based composites and compares their mechanical properties, thickness changes, and moisture absorption characteristics before and after artificial seawater exposure. Subsequently, the AHP method is employed to process the experimental data and determine the priority ranking of these composite materials for further applications.

## 2. Materials and Methodology

### 2.1. Materials and Fabrication Method

The current study uses three types of reinforcing fibers, including kenaf fiber, flax fiber, and glass fiber, whose mechanical properties are listed in Table 1. These fibers were used in the plain-weave form. The woven kenaf fabric had a thickness of 0.68 mm and an areal density of 275 g/m^2^ and was obtained from Lembaga Kenaf dan Tembakau Negara (LKTN), Kota Bharu, Malaysia. The woven flax fabric had a thickness of 0.40 mm and an areal density of 223 g/m^2^ and was obtained from Zhanjiang Xuesong Weaving Factory, China. Sodium hydroxide solution was used to treat both kenaf and flax fabric to remove surface impurities and enhance the adhesion between the fibers and the matrix [51,52]. The 5% NaOH solution was used to treat kenaf and flax fabrics at 25 °C for 4 h. In addition, the woven glass fabric had a thickness of 0.20 mm and an areal density of 220 g/m^2^ and was obtained from Shandong Fiberglass Group Co., Ltd., China. Employing glass fiber, especially in the form of outer wrapping fabrics, significantly improves the mechanical behavior of hybrid fiber-reinforced composites [53]. In addition, glass fibers have more stable properties and excellent environmental resistance compared to plant fibers. Moreover, a thermosetting epoxy resin system was used as the matrix for the manufacture of composites in this research because of its excellent mechanical properties, light weight, and chemical resistance [54]. The epoxy resin and curing agent were provided by IZE Solution SDN BHD, Malaysia. Moreover, the incorporation of nano-silica into FRCs has been proven to significantly enhance the overall performance of the materials [43,55]. The nano-silica used in this study had a bulk density of 0.14–0.18 g/cm^3^ and a specific surface area of 170–220 m^2^/g, and was supplied by Beesley New Materials (Suzhou) Co., Ltd., China. In addition, 3 wt.% nano-silica was incorporated into the epoxy in two separate steps, with 1.5 wt.% added in each step.

**Figure 1:**
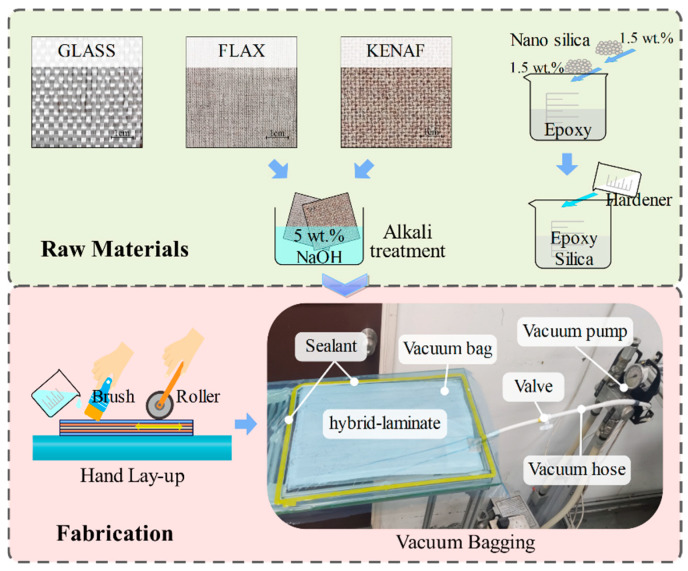
Fabrication of hybrid laminates.

**Figure 2:**
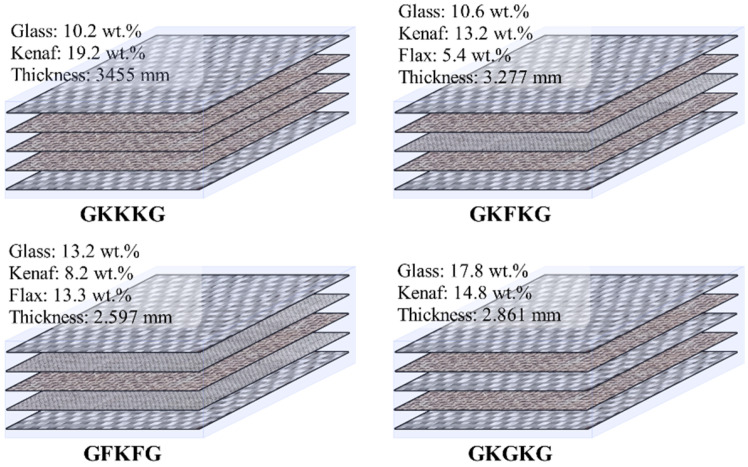
Five-layer hybrid fiber-reinforced laminates and their fiber content.

Hybrid laminates were fabricated using a composite technique combining hand lay-up and vacuum bagging methods with 400 × 300 mm dimensions. Combining the use of the vacuum bagging method can further eliminate voids, improve fiber-to–matrix adhesion, promote uniform resin distribution, and optimize fiber-to-resin ratios, significantly improving the quality of the composites when compared to the single use of traditional hand lay-up techniques [27]. Before fabricating laminates, the glass mold surface was cleaned and wiped with acetone repeatedly to ensure that all impurities were removed from the surface of the mold. After the mold surface dried, a uniform layer of release agent was applied on the mold to facilitate demolding and create a smooth composite surface. Subsequently, fiber fabric layers were placed onto the mold sequentially. The 3 wt.% nano-silica-enhanced matrix was evenly applied to the woven fiber layers using a brush and roller to ensure thorough impregnation. The roller was repeatedly used to gently compress the surface, eliminating trapped air and promoting full adhesion between the fibers and the matrix. Afterward, a sealing tape was placed around the mold perimeter, and a flexible vacuum bag was placed over the area. A vacuum pump was then used to evacuate the air, creating a sealed environment for further bonding and curing of the matrix and fibers, curing laminates at this condition for 24 h, as shown in Figure 1. Subsequently, the laminates were post-cured in an oven at 80 °C for 4 h. Finally, four types of composites were fabricated, and their naming and stacking sequence forms are shown in Figure 2, including GKKKG, GKFKG, GFKFG, and GKGKG. These laminates are a 5-layer symmetric structural design. The superior-performing glass fabrics are set up as the outer layer to isolate the environment and protect the inner layers. The core structure is formed by the interactive stacking of kenaf with flax and glass fibers.

### 2.2. Artificial Seawater Aging Process

Tensile and flexural specimens were cut from the fabricated composite laminates and subsequently soaked in artificial seawater at 25 °C. Their weights were measured at two-day intervals using a precision electronic balance. All specimens attained saturation after a 50-day immersion period. Artificial seawater was formulated in accordance with ASTM D1141, and the water absorption process complied with ASTM D570 standards [56,57]. The seawater absorption ratio (SWAR) and thickness swelling ratio (TSR) were calculated to assess the composites’ adaptability to the seawater environment. The SWAR indicates the moisture uptake behavior of composites in seawater, providing insights into their hydrophilicity. It is calculated using the following equation:

(1) $$SWAR\left(\%\right)=\frac{W_{t}-W_{0}}{W_{0}}\times 100$$

where $W_{0}$ denotes the initial mass of the specimen in a dry condition, and $W_{t}$ refers to the mass after immersion in seawater for a duration *t*.

The TSR quantifies the dimensional alteration in the thickness of the hybrid laminate following seawater immersion, serving as a critical indicator of dimensional stability under wet conditions. This value is calculated using the following formula:

(2) $$TSR(\%)=\frac{T_{t}-T_{0}}{T_{0}}\times 100$$

where $T_{0}$ and $T_{t}$ denote the initial thickness and the thickness after immersion in seawater for time *t*, respectively.

### 2.3. Tensile Testing

Tensile testing is generally conducted to evaluate the mechanical properties of materials under tensile loading and fracture morphology. Stress–strain curves are obtained to characterize the mechanical behavior of materials. Key tensile mechanical properties, such as ultimate tensile strength, tensile modulus, and elongation at break, are commonly derived through these tests. These parameters are crucial for material selection and engineering applications. In this study, uniaxial tensile tests were conducted in accordance with ASTM D3039 [58]. Standard specimens, prefabricated with dimensions of 250 mm in length and 25 mm in width, were tested using an MTS 810 servo-hydraulic testing machine under displacement-controlled conditions, with the crosshead speed maintained at 2 mm/min throughout the experiment.

### 2.4. Flexural Testing

Flexural testing is a fundamental technique for evaluating a material’s response to bending loads, providing critical data such as flexural strength and flexural modulus. These parameters are vital for assessing the load-bearing capacity of materials under flexural stress. According to ASTM D790 [59], three-point flexural tests were conducted using an MTS 810 testing system in the research. Rectangular specimens with a width of 13 mm were prepared for testing. During this test process, the crosshead speed was maintained at a constant rate of 2 mm/min, and loading continued until either the outer surface of the specimen fractured or the maximum strain reached 5.0%.

### 2.5. Analytic Hierarchy Process (AHP) Method

**Figure 3:**
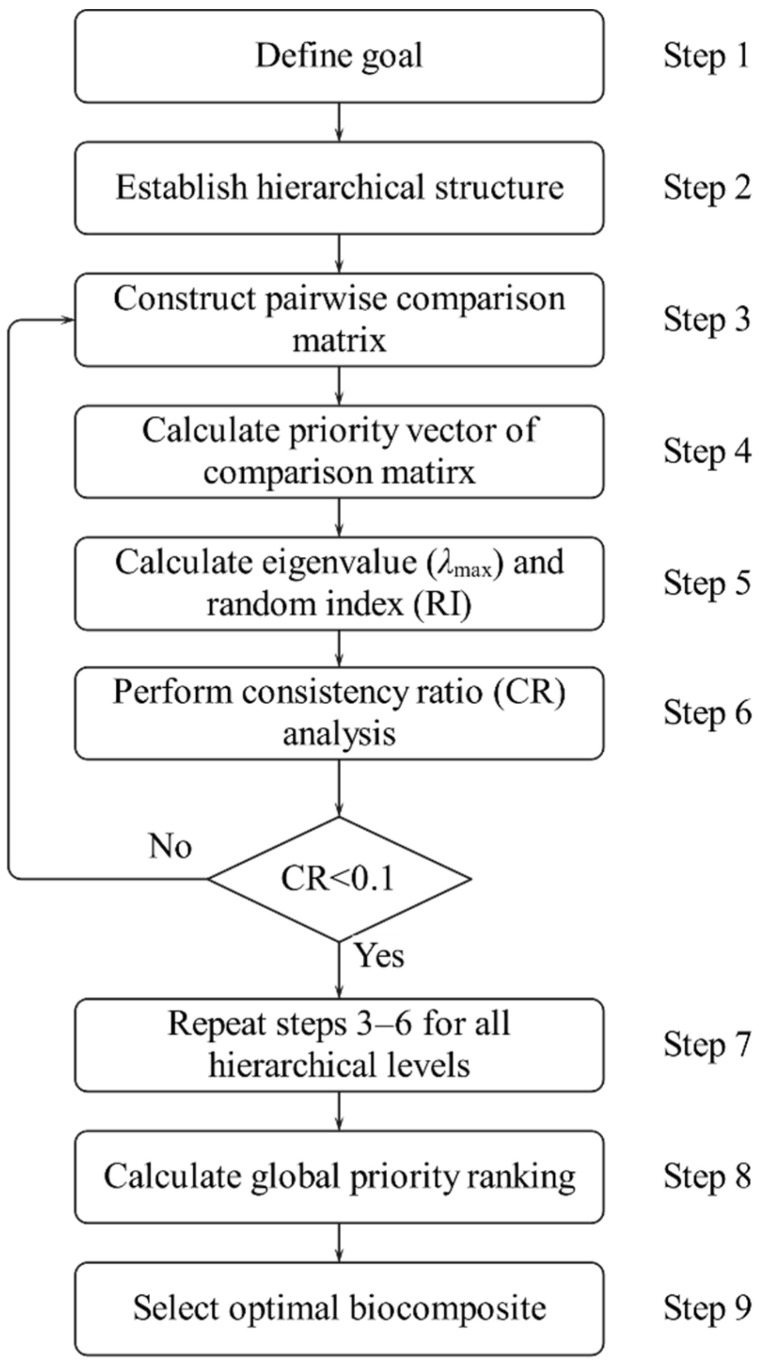
Flowchart of AHP.

Figure 3 illustrates the general flowchart of AHP, and the main process consists of 9 steps [47,50,60].

Step 1: The first step in applying the AHP is to define the decision problem explicitly and determine the primary objective. This involves identifying the relevant criteria that will guide the evaluation of alternatives within the decision-making framework.

Step 2: Constructing a hierarchical structure is a fundamental procedure of the AHP, as it clearly describes the relationships among the overall goal, main criteria, sub-criteria, and alternatives. This structured approach facilitates a comprehensive analysis of complex decision-making problems by organizing elements into a clear, multi-level framework.

Step 3: The third step in the AHP involves constructing a n×n pairwise comparison matrix, where n denotes the number of criteria or alternatives, as shown in Formula (3). This matrix is established by systematically comparing each criterion or alternative with all others. In addition, this matrix serves to clarify the relative importance of each criterion. When building pairwise comparisons, Saaty’s 1–9 scales are used in this study [47,60], as shown in Table 2.

(3) $$A_{n\times n}=\left[\begin{array}{cccc} a_{11} & a_{12} & \ldots & a_{1n}\\ a_{21} & a_{22} & \ldots & a_{2n}\\ \vdots & \vdots & \ddots & \vdots \\ a_{n1} & a_{n2} & \ldots & a_{nn} \end{array}\right]=\left[a_{ij}\right]$$

Steps 4 to 6: In the stages, the focus is on calculating the priority vector of the pairwise comparison matrix and verifying the consistency. These computations are carried out using the following mathematical formula:

The priority vector (*p_i_*) is calculated as follows:

(4) $$p_{i}=\frac{1}{n}\sum_{j=1}^{n}\;\frac{a_{ij}}{\sum_{i=1}^{n}\;a_{ij}},\;i,j=1,2,\cdots ,n$$

where p is a column vector, which refers to the priority vector of the pairwise comparison matrix $A_{n\times n}$, and $p_{i}$ is the *i*th component of p.

The maximum eigenvalue ($\lambda_{max}$) is calculated as follows:

(5) $$\lambda_{max}=\sum_{i=1}^{n}\;\frac{\sum_{j=1}^{n}\;a_{ij}\times p_{j}}{p_{i}},\;i,j=1,2,\cdots ,n$$

The consistency index (CI) is calculated as follows:

(6) $$CI=\frac{\lambda_{max}-n}{n-1}$$

The consistency ratio (CR) is calculated as follows:

(7) $$CR=\frac{CI}{RI}$$

where RI is the random index, as shown in Table 3.

Step 7: Steps 3 to 6 are repeated at each level of the hierarchical structure to calculate the priority vector for each standard or alternative.

Step 8: After performing the all-level consistency check, the overall priority vector is calculated to develop an overall rank.

Step 9: Based on the ranking derived from the previous step, the optimal material is selected.

## 3. Results and Discussion

### 3.1. Thickness Swelling and Seawater Uptake

**Figure 4:**
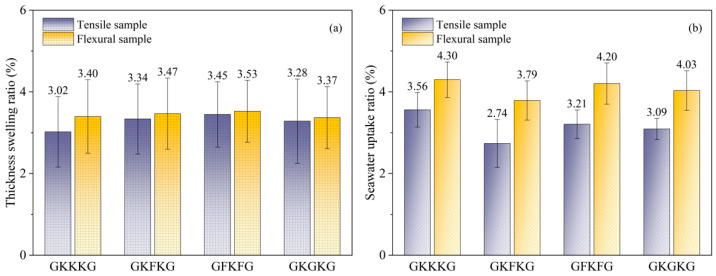
(**a**) Thickness swelling ratios of tensile and flexural specimens after seawater aging; (**b**) seawater uptake ratio of tensile and flexural specimens after seawater aging.

NFRCs exhibit water absorption behavior primarily due to the intrinsic hydrophilic nature of their constituent components. When exposed to an aqueous environment, composites absorb moisture directly from the surrounding water medium through surface interactions. The rate of water uptake is initially high but tends to gradually decrease as biocomposite materials approach a saturation state [61]. NFRCs are susceptible to degradation upon moisture exposure, primarily due to swelling and interfacial delamination [62]. When exposed to artificial seawater, swelling occurs along the thickness direction. The swelling ratios of the tensile and flexural specimens after 50 days of exposure in seawater are presented in Figure 4a. The thickness swelling ratios in the tensile samples for GKKKG, GKFKG, GFKFG, and GKGKG are 3.02%, 3.34%, 3.45%, and 3.28%, respectively. In addition, the flexural samples show slightly greater swelling, with corresponding values of 3.40%, 3.47%, 3.53%, and 3.37%, respectively. Compared to the tensile samples, the flexural samples exhibit greater thickness swelling deformation, increasing by 12.58%, 3.89%, 2.32%, and 2.74%, respectively. The results indicate that the thickness expansion ratio varies across different sample types, with smaller specimens being more susceptible to seawater-induced changes in thickness expansion. Among the tested composites, GKKKG exhibits the greatest increase in thickness swelling, whereas GFKFG shows the least, indicating superior dimensional stability of the latter. The stacking sequence and hybridization process significantly improve the interfacial adhesion between the fiber and matrix, effectively reducing the diffusion of water molecules within the composite material [63]. In addition, the use of low-hygroscopic materials in hybrid composites generates a barrier mechanism, effectively minimizing water absorption [17]. Furthermore, the standard deviation of laminate thickness variation is illustrated in Figure 4a. This variation can be attributed to two primary factors. First, the thickness change resulting from seawater absorption shows local non-uniformity within the composites. Second, the relatively thin nature of the tested laminates makes them susceptible to limitations in measurement precision.

The rate of water diffusion and the water absorption in hybrid composites are significantly influenced by factors such as fiber type, matrix composition, natural fiber weight content, and experimental temperature [35]. According to previous studies, these biocomposites reached an absorption equilibrium state within seven weeks of exposure to artificial seawater. Figure 4b presents the seawater absorption rates of tensile and flexural specimens following a continuous 50-day exposure to artificial seawater, revealing the long-term seawater uptake value of the composites. At the saturation stage, the seawater uptake ratios in the tensile specimens for GKKKG, GKFKG, GFKFG, and GKGKG are 3.56%, 2.74%, 3.21%, and 3.09%, respectively. In addition, the flexural samples show corresponding values of 4.30%, 3.79%, 4.20%, and 4.03%, respectively. The seawater absorption rate shows a positive relationship with plant fiber content. Moreover, altering stacking sequences changes the moisture diffusion pathways within composite materials, which has a mitigating effect on reducing the water absorption capability. Like the trend observed in thickness swelling, the flexural specimens exhibit a significantly higher weight increase, compared to the tensile specimens, by 20.79%, 38.32%, 30.84%, and 30.42%, respectively. These variations indicate that the composites exhibit greater sensitivity to seawater absorption compared to their dimensional changes in thickness in the same exposure environment [64]. Moreover, the dimensions of composite materials influence their water absorption behavior [65]. This indicates that geometric factors serve as a critical determinant in the moisture uptake behavior of composite materials. Furthermore, nanoparticles play a positive role in resisting water permeation. Liu et al. [42] investigated the effect of nano-silica interface modification on the water absorption behavior of jute/polypropylene composites. They found that the nanoparticles promoted molecular chain interlocking and polar interactions, which significantly inhibited water diffusion at the microscopic scale, therefore enhancing the composites’ water resistance and dimensional stability. Apart from these, a higher fiber reinforcement content leads to enhanced moisture absorption potential [10].

### 3.2. Tensile Properties

**Figure 5:**
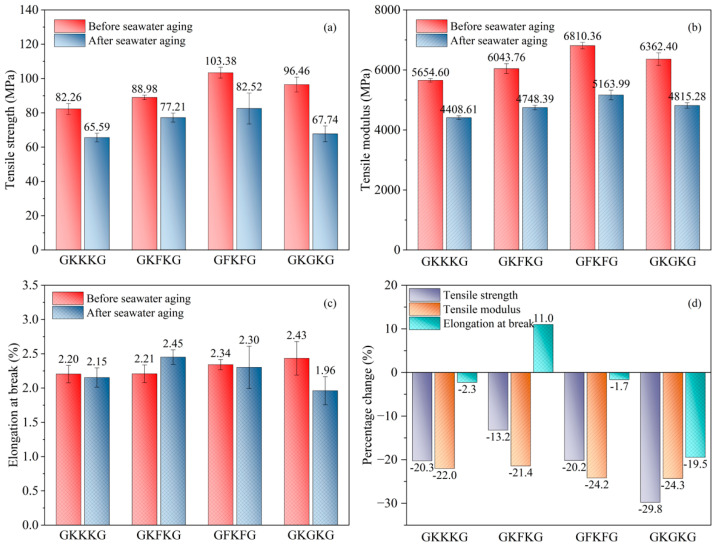
(**a**) Tensile strength of composites before and after seawater aging; (**b**) tensile modulus of composites before and after seawater aging; (**c**) elongation at break of composites before and after seawater aging; (**d**) percentage variation in tensile properties of composites before and after seawater aging.

The tensile test results of the four composites in both dry and seawater-saturated states are shown in Figure 5. Regardless of whether the composites are in a dry state or saturated with seawater, their tensile properties are significantly influenced by the stacking sequence. In Figure 5a, increasing the glass fiber content in GKGKG improves its tensile performance beyond that of GKKKG and GKFKG but still lags behind that of GFKFG. GFKFG displays the highest tensile strength performance under both dry (103.38 MPa) and seawater-saturated (82.52 MPa) conditions, which can be primarily attributed to the superior tensile properties of flax fibers, as the dual flax fiber layer in GFKFG provides enhanced load-bearing capability. The finding indicates altering the stacking sequence can significantly modify mechanical behaviors. Furthermore, GFKFG maintains a relatively higher proportion of cellulose fibers in the tested composite materials. In contrast, GKKKG shows the lowest tensile strength under both dry (82.26 MPa) and seawater-saturated (65.59 MPa) conditions. As shown in Figure 5b, GFKFG consistently demonstrates the highest tensile modulus under both dry and seawater-treated conditions, which aligns with its superior performance in terms of tensile strength. This finding demonstrates that the composite material incorporating kenaf, flax, and glass fibers possesses highly valuable mechanical properties. In contrast, GKKKG consistently exhibits the lowest tensile modulus across both tested states. The results demonstrate that hybridizing the different plant fabrics at the inner layer of laminates can effectively strengthen the mechanical properties of the composite materials and improve their resistance to seawater aging. Moreover, these figures reveal that seawater aging induces a degradation in tensile strength, modulus, and elongation at break of the composites, highlighting the negative impact of moisture ingress on their tensile performances. During seawater aging, the matrix, reinforcements, and their interfaces suffer from gradual degradation, and then the composites are deteriorated under the dual action of physical exfoliation and chemical erosion [66]. However, it is noteworthy that GKFKG shows an increasing trend in elongation at break after seawater aging, as shown in Figure 5c, indicating that the effects of seawater aging on elongation at break vary across different hybrid laminates [67].

Figure 5d illustrates the specific impact of seawater aging on tensile properties. After seawater aging treatment, the tensile strength of GKKKG, GKFKG, GFKFG, and GKGKG declines by 20.3%, 13.2%, 20.2%, and 29.8%, respectively. A similar trend is observed in tensile modulus, which decreases by 22.0%, 21.4%, 24.2%, and 24.3%. In elongation at break, GKKKG, GFKFG, and GKGKG show respective reductions of 2.3%, 1.7%, and 19.5%. Interestingly, GKFKG is the only composite to display a positive change, with an 11.0% increase in elongation, indicating the stacking sequence significantly influences the variations in elongation and ductility of composites after seawater absorption. Moreover, the presence of water molecules in the saturated composites may have a positive impact on increasing the fracture elongation rate [67]. Chow et al. [68] obtained a similar finding. They investigated the moisture absorption behavior of sisal-fiber-reinforced polypropylene composites and revealed that water uptake induced a plasticizing effect on both the sisal fibers and the fiber–matrix interface, which, in turn, increased the material’s elongation at break under tensile loading. In addition, seawater aging causes matrix degradation and a reduction in fiber–matrix interfacial bonding, thereby decreasing the stiffness of composites and altering their tensile performance [33].

Seawater, rich in salts and corrosive chemical components, accelerates the degradation of biocomposites [20]. For instance, Fiore et al. [69] evaluated the aging resistance of flax and jute-fiber-reinforced epoxy resin composites in marine environments and indicated that salt spray aging progressively deteriorated the tensile and flexural properties of the composites due to the erosion of interfacial bonding between fibers and matrix. Moreover, they also reported that composites with treated flax fibers and epoxy resin exhibited improved interfacial compatibility, which explains the positive effect of incorporating flax fibers in composites to enhance the mechanical properties. In addition, due to the uneven quality and porosity of NFRCs, there is a risk of local saturation, especially at the interface between the matrix and reinforcement, which leads to uneven water absorption and adversely affects mechanical properties [33]. According to Guo et al. [21], water exposure causes fiber/resin interface debonding, forming weak layers that provide additional paths for water diffusion. Moreover, the plasticization of the polymer matrix and swelling of the laminate in seawater environments lead to the degradation and weakening of the fiber–matrix interface, ultimately causing a reduction in the mechanical properties of seawater-aged laminates [70,71].

**Figure 6:**
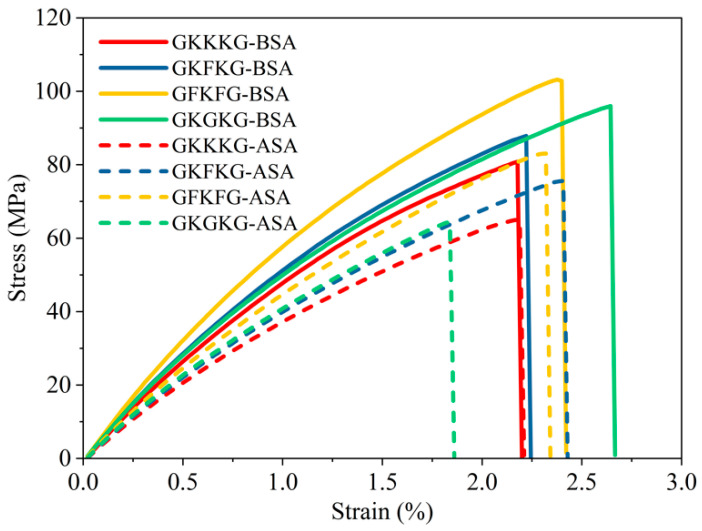
Tensile stress–strain curves of composites before and after seawater aging.

Figure 6 shows the typical tensile stress–strain curves of the four composites under both dry and seawater-saturated conditions. The seawater-aged specimens, like those in the dry state, display a progressive linear relationship between stress and strain during the initial phase of tensile loading. As the tensile loading progresses, all the curves exhibit a transition from linear to nonlinear behavior, followed by a sharp vertical drop when reaching the peak point, signifying material fracture and loss of load-bearing capacity. The observed characteristics of the curves are consistent with the findings reported by Zakaria et al. [72]. In addition, the morphology of the stress–strain curves reveals that the failure of these composite specimens is brittle and occurs abruptly [73]. Moreover, although variations in stacking patterns and sample conditions (dry or wet) influence the evolution of the stress–strain curves, they do not fundamentally change the damage mechanisms of the composites.

Table 4 presents the tensile strength, tensile modulus, and their respective retention rates of the composites before and after artificial seawater aging. Among the four material configurations, GKFKG demonstrates the highest retention rates of tensile strength (86.77%) and modulus (78.57%) after seawater aging. This result can be attributed to the incorporation of flax fiber in the hybrid composite, which increases the resistance to seawater degradation. Conversely, GKGKG has the worst performance, with retention rates of tensile strength and modulus of 70.23% and 75.68%, respectively. Therefore, the findings indicate that the arrangement of the glass fabric as an interlayer for seawater application is not an optimal material choice in these terms. Nevertheless, separating glass fibers from the polyester matrix requires more energy compared with natural fibers; moreover, the arrangement of the glass fiber layer as the surface layer has a positive effect on resisting external influences [74].

### 3.3. Flexural Properties

**Figure 7:**
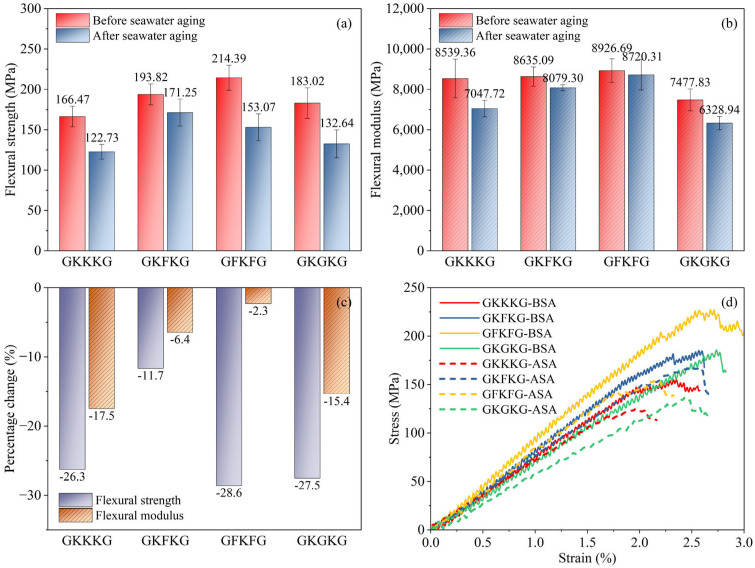
(**a**) Flexural strength of composites before and after seawater aging; (**b**) flexural modulus of composites before and after seawater aging; (**c**) percentage variation in flexural properties of composites before and after seawater aging; (**d**) flexural stress–strain curves of composites before and after seawater aging.

The flexural properties of the composites before and after seawater aging are shown in Figure 7. Average critical flexural behavior data for each hybrid composite is recorded and analyzed in this section. As illustrated in Figure 7a, the initial flexural strength values of GKKKG, GKFKG, GFKFG, and GKGKG are measured at 166.47, 192.82, 214.39, and 183.02 MPa, respectively, while after 50-day artificial seawater aging, the corresponding values decrease to 122.73, 171.25, 153.07, and 132.64 MPa. In the dry state, the flexural strength value of GFKFG is the highest, while that of GKKKG is the lowest. In contrast, GFKFG has the highest flexural strength value after seawater aging, and GKKKG still displays the lowest value. Additionally, in Figure 7b, the flexural modulus of GFKFG is the highest (8926.69 MPa) in the dry state, while that of GKGKG is the lowest composite. In contrast, GFKFG has the highest flexural modulus value (8720.31 MPa) after seawater aging, and GKGKG has the lowest one. The variation in flexural performance is primarily attributed to disparities in the material properties of individual fiber layers, modifications in the stacking sequence, and changes in laminate thickness induced by the altered layer configuration.

Figure 7c presents a comparative analysis of the percentage changes in flexural strength and modulus for each composite before and after seawater aging, providing intuitive data on the degradation of the material’s flexural behavior. GFKFG exhibits the highest reduction (28.6%) in flexural strength after seawater aging. In contrast, its flexural modulus declines by only 2.3%, which is the smallest change among the four composites. This finding indicates that seawater aging differentially impacts flexural strength and modulus, highlighting the necessity of assessing multiple mechanical properties to comprehensively evaluate material durability. Moreover, GKFKG had the lowest reduction (11.7%) in flexural strength after seawater aging, indicating that it has a better strength preservation rate in the marine environment and possesses extremely potential high application value in this condition. The variations in flexural degradation reflect the differing impacts of seawater on the interfacial bonding strength between the fibers and the matrix. Additionally, moisture uptake from the seawater environment degrades the flexural performance of composites, increasing vulnerability due to matrix fracture, interfacial debonding, and delamination [75].

The mass fraction and arrangement pattern of the fibers and resin, as well as the manufacturing process of the composites, have a direct influence on the mechanical properties of the hybrid composites [27]. In addition, modified nanoparticles enhance the bonding properties between fibers and polymers and have a positive effect on the bending properties of materials [76]. According to previous research, mixing appropriate concentrations of silica into FRCs can strengthen the interfacial bonding between fibers and the matrix and reduce fiber pull-out and delamination [44,55,77]. This modification not only significantly enhances the overall performance of composite materials but also effectively reduces moisture absorption. However, after aqueous environment exposure, the moisture ingress into composite materials weakens their internal chemical bonds and interfacial interactions, promoting void formation in their internal location [35]. These voids may further induce internal and surface microcracks, ultimately diminishing material mechanical performance [35]. The curves, as shown in Figure 7d, illustrate the stress–strain relationships for the four composites in the dry and seawater-saturated states. The stress–strain curves of seawater-aged composites fall below those of the same composites in the dry state. However, the curves of these seawater-aged composites can still maintain their morphological characteristics in the dry state.

Thus, rational design of fiber-reinforced composite materials has a positive effect on improving mechanical properties and minimizing the impact of seawater aging. Hybridization technology generates synergistic interactions among components, offsetting the inherent limitations of individual reinforcing fibers and producing hybrid fiber composites with enhanced mechanical and durability characteristics [8].

Table 5 presents the tested composites’ flexural strength, flexural modulus, and retention rates before and after artificial seawater aging. GKFKG exhibits the highest retention of flexural strength (88.36%), while GFKFG shows the highest retention of flexural modulus (97.69%), highlighting the reinforcing effectiveness of flax fabric in the laminate structure. Conversely, GKGKG demonstrates the lowest flexural strength retention (72.47%), and GKKKG shows the lowest retention rates of flexural modulus (82.53%). Thus, rational design of fiber-reinforced composite materials has a positive effect on improving mechanical properties and minimizing the impact of seawater aging. Hybridization technology generates synergistic interactions among components, offsetting the inherent limitations of individual reinforcing fibers and producing hybrid fiber composites with enhanced mechanical and durability characteristics [8].

### 3.4. AHP Analysis

**Figure 8:**
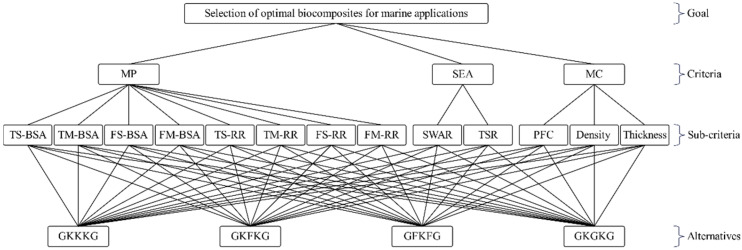
Hierarchical structure of AHP.

The establishment of an appropriate AHP framework is vital for achieving accurate and consistent decision-making results. Typically, developing NFRCs for structural applications is governed by several primary design parameters, including mechanical performance, environmental adaptability, physical characteristics, and sustainability. A four-level hierarchical framework was established for the selection of optimal composites for marine applications, as shown in Figure 8. The first level of the hierarchy structure is the overall goal. Based on this goal, three main criteria, such as mechanical performance (MP), seawater environmental adaptability (SEA), and material characteristics (MC), are defined in the second level. In the third level, thirteen sub-criteria are used, covering mechanical and physical properties of alternatives, such as tensile strength before seawater aging (TS-BSA), tensile modulus before seawater aging (TM-BSA), flexural strength before seawater aging (FS-BSA), flexural modulus before seawater aging (FM-BSA), and their corresponding retention rates after seawater aging, including retention rate of tensile strength after aging (TS-RR), retention rate of tensile modulus after aging (TM-RR), retention rate of flexural strength after aging (FS-RR), retention rate of flexural modulus after aging (FM-RR), seawater absorption rate (SWAR), thickness swelling ratio (TSR), plant fiber content (PFC), density, and thickness. Finally, four composite alternatives are evaluated at the fourth level to rank their suitability for UMAs applications.

Based on the hierarchical framework, the relevant composite data for AHP analysis is collected and summarized in Table 6. The increase in mechanical properties is favorable for these composites. However, the increases in SWAR and TSR, which negatively affect the stability of the composites, are classified as negative criteria. Additionally, increased density and thickness are undesirable for lightweight design and are classified as negative criteria in this analysis. The values presented in this table will be used to construct the pairwise comparison matrix; however, the data corresponding to negative criteria needs to be processed prior to matrix formation. The values of alternatives associated with negative priorities are transformed into positive priorities by applying the reciprocal transformation (*a_ij_* = 1/*a_ij_*) [78,79].

**Figure 9:**
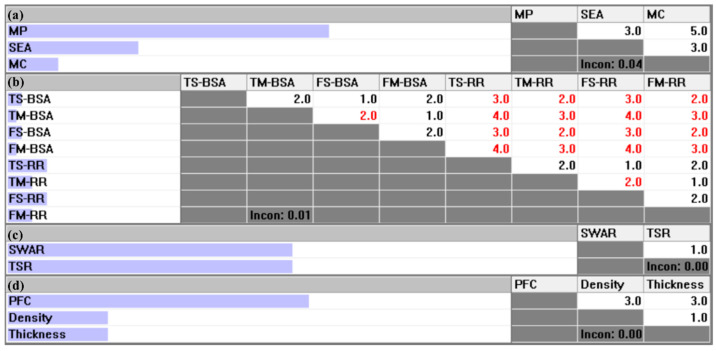
Pairwise comparison matrices of criteria: (**a**) local priorities of main criteria with respect to the overall goal; (**b**) local priorities of sub-criteria under the first-level criterion MP; (**c**) local priorities of sub-criteria under the first-level criterion SEA; (**d**) local priorities of sub-criteria under the first-level criterion MC.

The selection process was implemented using Expert Choice 11.5 software. Expert Choice is decision-support software developed based on the AHP methodology for multi-attribute decision-making tasks [80,81]. Hambali et al. [57] successfully applied the AHP method with Expert Choice to select suitable materials for polymeric composite automotive bumper beams. Similarly, this software was also adopted by Naveen et al. [49] and Balakrishnan et al. [50] to rank natural fibers for fabricating hybrid composite materials. Figure 9a–d presents the pairwise comparison matrices at each hierarchy criterion level. Considering the critical role of material properties for structural safety when employed in UMAs, the main criteria are prioritized in the following order: MP>SEA>MC. Moreover, the sub-criteria are prioritized within each main criterion as follows: for MP, TS-RR=FS-RR>TM-RR=FM-RR>TS-BSA=FS-BSA>TM-BSA=FM-BSA; for SEA, SWAR=TSR; and for MC, PFC>Density=Thickness. In these matrices, black-colored values indicate that the row element is assigned a higher priority than the column element, while red-colored values signify the opposite. This rule is consistently applied across all levels of the hierarchy in the software. Moreover, as shown in Figure 9, all pairwise comparison matrices present acceptable consistency, with CR below the 0.10 threshold [60].

**Figure 10:**
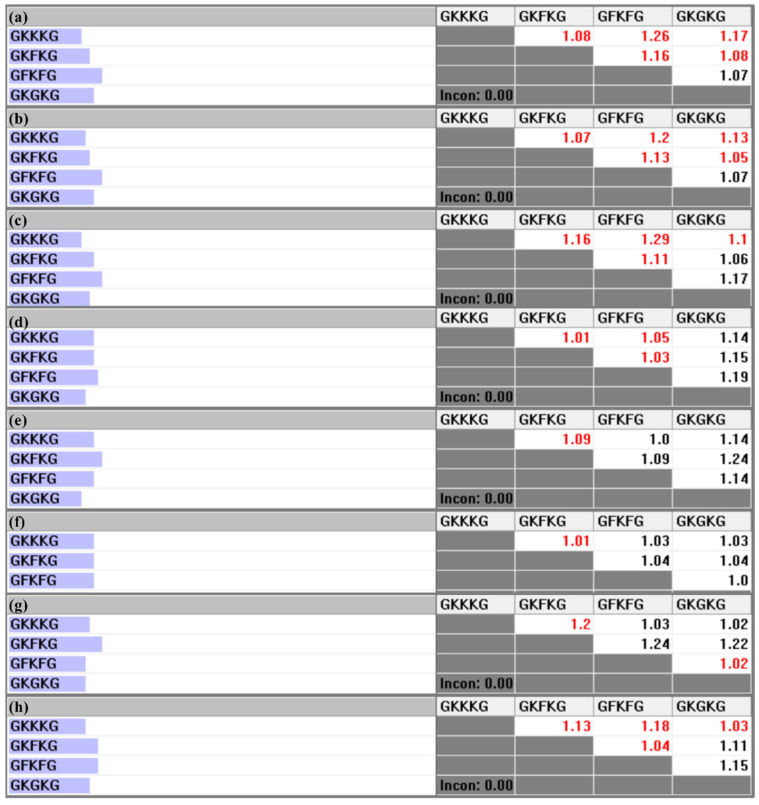
Pairwise comparison matrices of alternatives under the sub-criteria of MP: (**a**) relative importance of alternatives with respect to the TS-BSA; (**b**) relative importance of alternatives with respect to the TM-BSA; (**c**) relative importance of alternatives with respect to the FS-BSA; (**d**) relative importance of alternatives with respect to the FM-BSA; (**e**) relative importance of alternatives with respect to the TS-RR; (**f**) relative importance of alternatives with respect to the TM-RR; (**g**) relative importance of alternatives with respect to the FS-RR; (**h**) relative importance of alternatives with respect to the FM-RR.

**Figure 11:**
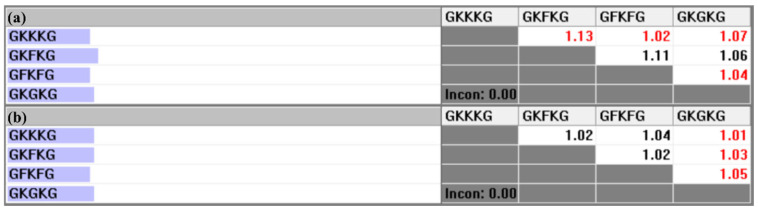
Pairwise comparison matrices of alternatives under the sub-criteria of SEA: (**a**) relative importance of alternatives with respect to the SWAR; (**b**) relative importance of alternatives with respect to the TSR.

**Figure 12:**
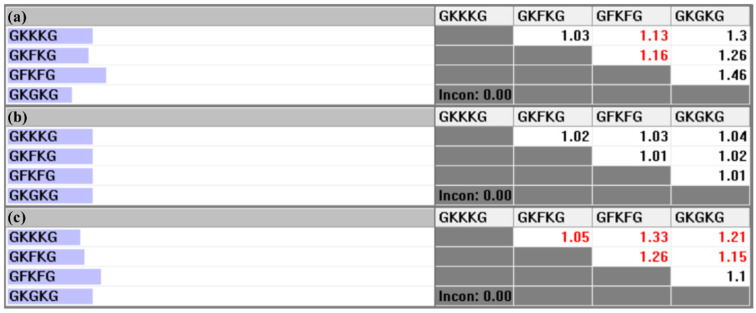
Pairwise comparison matrices of alternatives under the sub-criteria of MC: (**a**) relative importance of alternatives with respect to the TS-BSA; (**b**) relative importance of alternatives with respect to the TM-BSA; (**c**) relative importance of alternatives with respect to the FS-BSA.

The pairwise comparison matrix of the alternatives with respect to each criterion is constructed based on the parameter data collected for each composite, as presented in Table 6. Figure 10, Figure 11, and Figure 12 present the pairwise comparison matrices of the evaluated alternatives with respect to the sub-criteria under MP, SEA, and MC, respectively. The relative importance scale values in each pairwise comparison matrix are derived by calculating the ratio of the property values exhibited by each alternative [50]. For instance, as shown in Table 6, the TS-BSA values for GFKFG and GKGKG are 103.38 MPa and 96.46 MPa, respectively. By computing the ratio (103.38/96.46), a relative importance scale value of 1.07 is obtained in Figure 10a, where the use of black color indicates that GFKFG exhibits superior performance to GKGKG in this criterion. Furthermore, the FS-BSA values of GKKKG and GKFKG in Table 6 are 166.47 MPa and 193.82 MPa, respectively. Calculating their ratio (166.47/193.82) leads to a relative importance scale value of 0.86, indicating that GKKKG demonstrates inferior performance to GKFKG under this criterion. Then, in Figure 10c, the reciprocal value of 0.86 is 1.16, used with the red color that highlights the superior ranking of GKFKG over GKKKG. The resulting values from this calculation are employed to formulate the pairwise comparison matrices utilized in the AHP [79].

**Figure 13:**
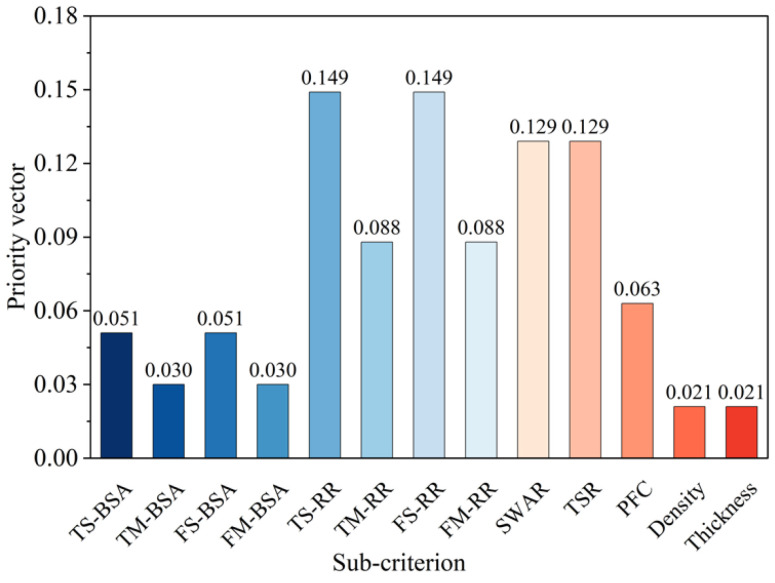
Synthesis priority vectors of sub-criteria with respect to overall goal.

Within the AHP, each sub-criterion exerts an indirect influence on the overall goal through its corresponding upper-level criterion. In the two-level criteria hierarchy, the global influence of each sub-criterion is determined by multiplying its local weight by the global weight of its associated parent criterion [82]. The priority vector results for the sub-criteria relative to the overall goal are presented in Figure 13. TS-RR and FS-RR exhibit the highest contributions, with priority vectors of 0.149, while density and thickness show the lowest contributions, with a priority vector of 0.021.

**Figure 14:**
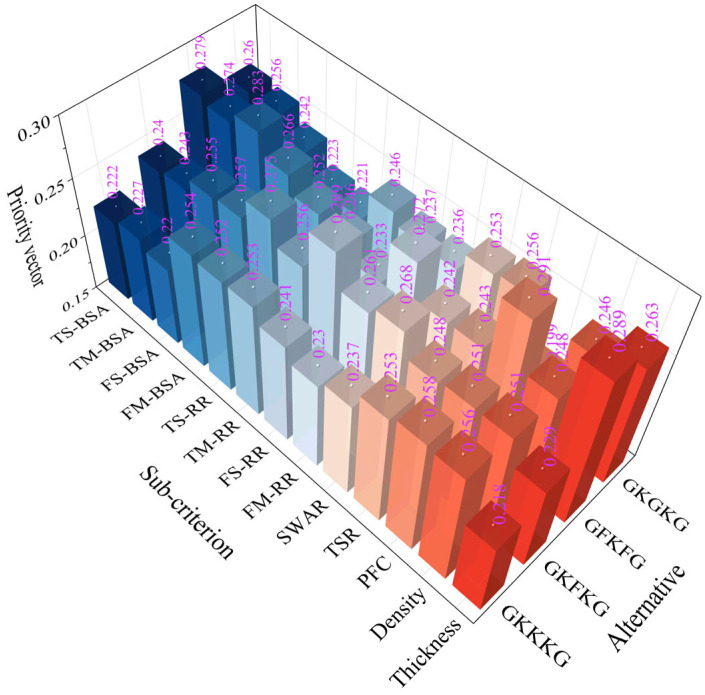
Synthesized priorities of alternatives with respect to each sub-criterion.

Figure 14 illustrates the priority vectors of each alternative with respect to the sub-criteria under MP, SEA, and MC. The alternatives show distinct preference levels with respect to the evaluated sub-criteria. For instance, GFKFG demonstrates outstanding overall performance, achieving the highest rankings across seven evaluation criteria, such as TS-BSA, TM-BSA, FS-BSA, FM-BSA, FM-RR, PFC, and thickness, indicating its broad dominance in both mechanical and physical characteristics. In addition, GKFKG demonstrates excellence in four specific criteria, namely, TS-RR, TM-RR, FS-RR, and SWAR, highlighting its relative advantage in retention-related characteristics and resistance to seawater absorption. In contrast, GKKKG and GKGKG exhibit notable advantages solely in terms of density and TSR, respectively, indicating that these two composites possess limited performance benefits and a narrow scope of superiority when compared to both GKFKG and GFKFG.

**Figure 15:**
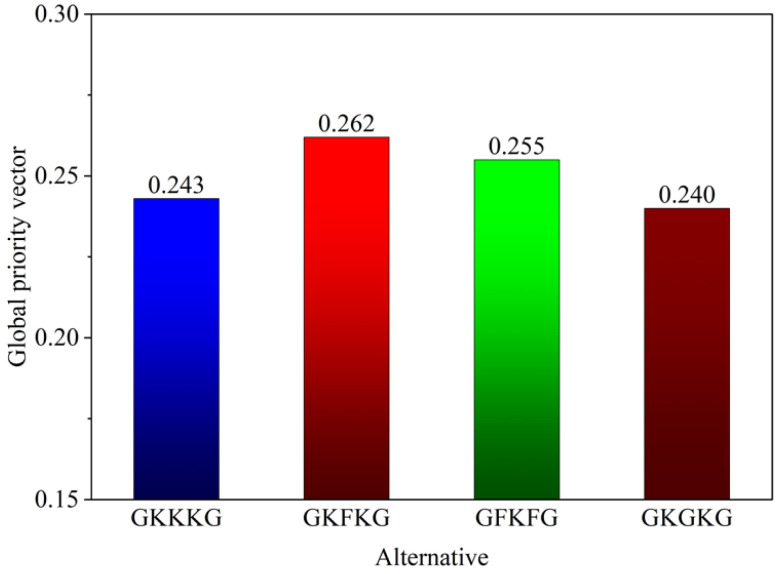
Overall synthesis of global priority vectors for all alternatives based on the AHP.

Figure 15 illustrates the global priority vector calculated for each alternative composite referring to the established evaluation criteria. Based on the global priority vector illustrated in the figure, the four composites are ranked in the following descending order: GKFKG>GFKFG>GKKKG>GKGKG. GKFKG, achieving the highest priority vector of 0.262, is identified as the most appropriate hybrid composite for application in marine environments. The result is primarily attributed to the superior mechanical performance retention rate of GKFKG after seawater aging, which reflects the more stable resistance of the composite to marine degradation compared to other samples. In the AHP calculation process, the overall consistency ratio was recorded as 0.04, and all pairwise comparison matrices exhibited consistency ratios below the 0.1 threshold, confirming that the judgments made during the procedure are consistent and reliable [50].

In the MCDM process, sensitivity analysis is crucial for understanding how changes in criterion weights affect the final ranking of alternatives, and it serves as an effective approach for assessing the stability of the results [83]. In addition, the AHP method suffers from the impact of subjective judgments; therefore, detecting the stability of the ranking under different weights of the criteria is essential [60]. The analysis can be performed by increasing or decreasing the weight of each criterion to check the change in the prioritization of alternatives. The Expert Choice system displays an excellent performance in this work [49,50]. This process evaluates the influence of each special criterion weight on the overall decision outcome and analyzes the stability and reliability of the AHP. Therefore, the variation in the ranking of alternatives associated with each criterion is examined by increasing the weight of each criterion by 25% to assess the sensitivity of the AHP [49,60].

**Figure 16:**
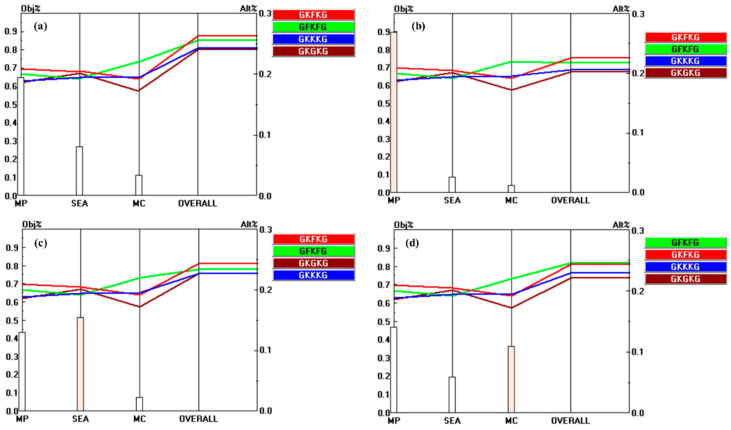
Performance sensitivity analysis of the main criteria with respect to the overall goal: (**a**) initial condition; (**b**) after increasing the weight of MP by 25% (from 63.7% to 88.7%); (**c**) after increasing the weight of SEA by 25% (from 25.8% to 50.8%); (**d**) after increasing the weight of MC by 25% (from 10.5% to 35.5%).

**Figure 17:**
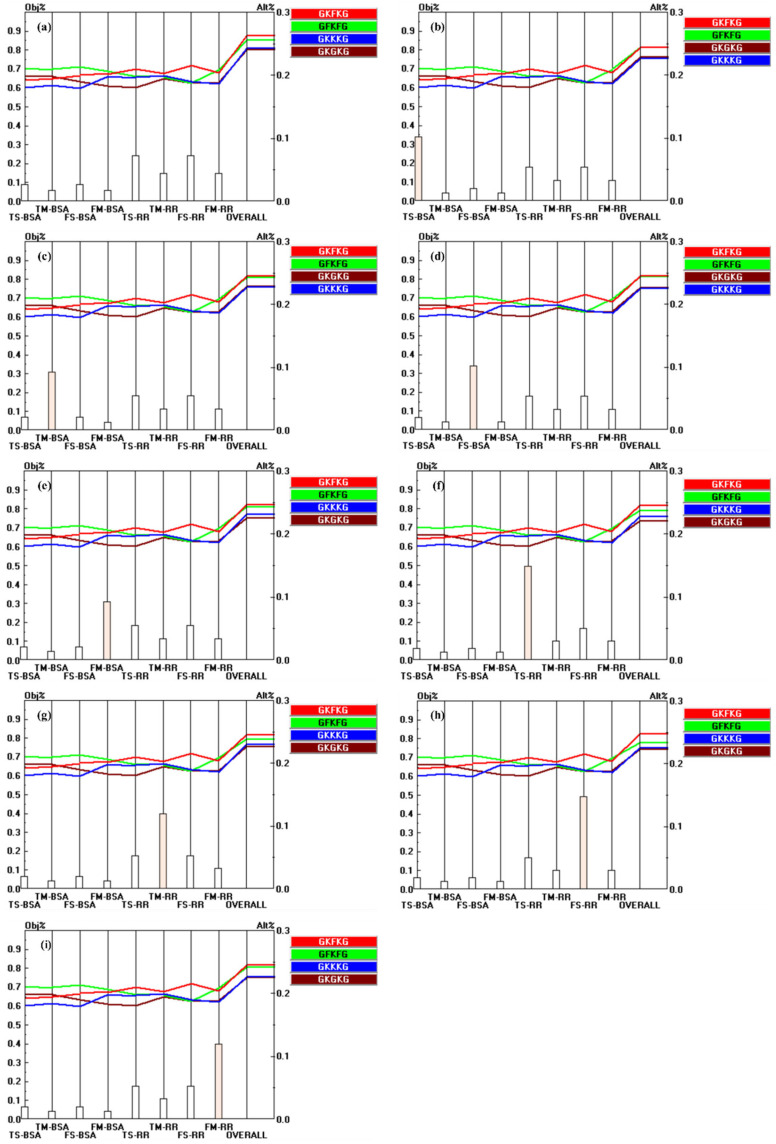
Performance sensitivity analysis of the sub-criteria under MP with respect to the overall goal: (**a**) initial condition; (**b**) after increasing the weight of TS-BSA by 25% (from 8.0% to 33.0%); (**c**) after increasing the weight of TM-BSA by 25% (from 4.8% to 29.8%); (**d**) after increasing the weight of FS-BSA by 25% (from 8.0% to 33.0%); (**e**) after increasing the weight of FM-BSA by 25% (from 4.8% to 29.8%); (**f**) after increasing the weight of TS-RR by 25% (from 23.4% to 48.4%); (**g**) after increasing the weight of TM-RR by 25% (from 13.9% to 38.9%); (**h**) after increasing the weight of FS-RR by 25% (from 23.4% to 48.4%); (**i**) after increasing the weight of FM-RR by 25% (from 13.9% to 38.9%).

**Figure 18:**
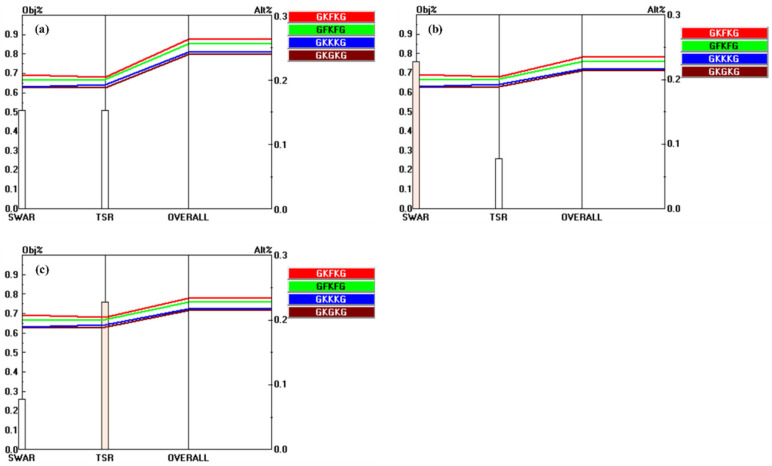
Performance sensitivity analysis of the sub-criteria under SEA with respect to the overall goal: (**a**) initial condition; (**b**) after increasing the weight of SWAR by 25% (from 50.0% to 75.0%); (**c**) after increasing the weight of TSR by 25% (from 50.0% to 75.0%).

**Figure 19:**
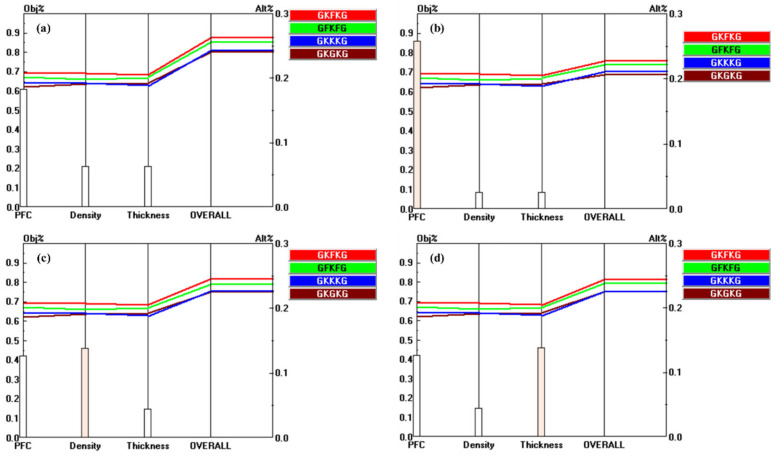
Performance sensitivity analysis of the sub-criteria under MC with respect to the overall goal: (**a**) initial condition; (**b**) after increasing the weight of PFC by 25% (from 60.0% to 85.0%); (**c**) After increasing the weight of density by 25% (from 20.0% to 45.0%); (**d**) after increasing the weight of thickness by 25% (from 20.0% to 45.0%).

Figure 16 illustrates the outcomes of the sensitivity analysis, highlighting the changes in both the priority values and ranking positions of the alternatives in relation to the criteria MP, SEA, and MC, under initial conditions and following a 25% increase in the respective criterion weights. Increasing the weights of SEA and MC has a substantial impact on the final ranking of alternatives. Specifically, a 25% increase in the weight of the MC criterion leads to a significant shift in the ranking of the top alternative, underscoring its substantial impact on the decision-making outcome. This result indicates that GFKFG has higher sensitivity under the MC set of criteria, which is consistent with the performance of the GFKFG priority vector shown in Figure 14. In addition, Figure 17, Figure 18, and Figure 19 present the performance sensitivity analyses of sub-criteria under MP, SEA, and MC, respectively, in association with the overall goal. The figures show that the ranking of the alternatives undergoes changes only when the weights of TS-BSA, TM-BSA, and FS-BSA are increased by 25%, highlighting that the three criteria exhibit strong sensitivity in the process compared to the others in the AHP. The ranking and priority values of the composites are presented in Table 7 after a 25% increase in the weights of SEA, MC, TS-BSA, TM-BSA, and FS-BSA.

The sensitivity analysis results reveal that GKFKG and GFKFG consistently rank first and second, respectively. This analysis effectively evaluates the result stability for making the best decisions [83,84]. Although a 25% increase in the weights of the TS-BSA, TM-BSA, and FS-BSA criteria results in ranking adjustments, the changes are limited to the third- and fourth-ranked alternatives. These findings demonstrate the reliability and consistency of the AHP method in evaluating composite materials for marine applications. The sensitivity analysis further highlights the beneficial role of incorporating kenaf, flax, and glass fibers as reinforcements in enhancing the seawater resistance of hybrid composites.

While this study provides valuable insights, several limitations exist that need to be considered. For example, only four five-layer kenaf/flax/glass hybrid laminates were investigated for tensile, flexural, and thickness properties before and after seawater exposure. In addition, the AHP method relied only on current limited experimental data. Therefore, further research is needed to advance cellulose-fiber-based composite applications in marine environments.

## 4. Conclusions

This study compared the changes in thickness, tensile, and flexural properties of hybrid composite materials after artificial seawater exposure. Subsequently, the AHP was employed to determine the optimal ranking of these composite materials for marine environment applications. The key findings of this research are summarized as follows:

1. Seawater immersion negatively impacted the mechanical properties of composite materials. After seawater aging, GFKFG exhibited the highest tensile strength and tensile modulus, while GKFKG showed the greatest elongation. In addition, GFKFG exhibited the highest flexural strength and flexural modulus, while GKGKG showed the greatest reduction in flexural properties.
2. Following the AHP analysis, GKFKG emerged as the optimal alternative, fulfilling both the global objectives and operational criteria, while the remaining alternatives were ranked in descending order: GFKFG, GKKKG, and GKGKG.
3. The consistency ratios for all pairwise comparison matrices are below the acceptable threshold of 0.1, confirming the reliability and validity of the AHP results.
4. The sensitivity analysis further demonstrated the stability of the AHP method. GKFKG maintained the highest priority in most scenarios following a 25% increase in criterion weights, while GFKFG led the ranking for the sub-criteria TS-BSA, TM-BSA, and FS-BSA. These results also highlight the positive synergistic effect of kenaf, flax, and glass fibers as reinforcing fibers in the composites.

In summary, these findings have revealed the potential application value of hybrid composite materials in the field of UMVs. This research establishes a significant foundation for advancing kenaf/flax/glass-fiber-based composites in marine environments. Additionally, the AHP method demonstrates significant advantages in multi-criteria decision-making while maintaining high credibility. Moreover, the experimental methods and material selection strategies involved in this study also have a positive effect on promoting the broader industrial application of hybrid composite materials.

## Figures and Tables

**Table 1:** Mechanical properties of reinforcing fibers [8] and epoxy resin system.

Materials	Tensile Strength (MPa)	Young’s Modulus (GPa)	Elongation at Break (%)	Density (g/cm^3^)
Kenaf fiber	930	53	1.6	1.2
Flax fiber	1400	70	1.6	1.4
Glass fiber	1950	72	2.7	2.55
Epoxy	54.5	3.1	2.6	1.1

**Table 2:** Fundamental scale for pairwise comparisons in AHP [47,60].

Relative Intensity Scale	Definition	Explanation
1	Equal importance	Two factors are equally important.
3	Slightly more importance	Experience slightly favors one factor over the other.
5	Strong importance	Experience strongly favors one factor over the other.
7	Very strong importance	One factor is strongly favored, and its dominance is demonstrated in practice.
9	Extreme importance	Evidence favoring one over another is of the highest possible order of affirmation.
2, 4, 6, 8	Intermediate value between two adjacent judgments	Applied when compromise is needed.

**Table 3:** Random index of AHP [50,60].

Matrix Size *n*	1	2	3	4	5	6	7	8	9	10
RI	0	0	0.58	0.90	1.12	1.24	1.32	1.41	1.45	1.49

**Table 4:** Tensile strength, tensile modulus, and respective retention rate.

Type of Composite	Average Tensile Strength (MPa)		Retention Rate of Strength (%)	Average Tensile Modulus (MPa)		Retention Rate of Modulus (%)
	Before	After		Before	After	
GKKKG	82.26	65.59	79.73	5654.60	4408.61	77.97
GKFKG	88.98	77.21	86.77	6043.76	4748.39	78.57
GFKFG	103.38	82.52	79.82	6810.36	5163.99	75.83
GKGKG	96.46	67.74	70.23	6362.40	4815.28	75.68

**Table 5:** Flexural strength, flexural modulus, and respective retention rate.

Type of Composite	Average Flexural Strength (MPa)		Retention Rate of Strength (%)	Average Flexural Modulus (MPa)		Retention Rate of Modulus (%)
	Before	After		Before	After	
GKKKG	166.47	122.73	73.72	8539.36	7047.72	82.53
GKFKG	192.82	171.25	88.36	8635.09	8079.3	93.56
GFKFG	214.39	153.07	71.40	8926.69	8720.31	97.69
GKGKG	183.02	132.64	72.47	7477.83	6328.94	84.64

**Table 6:** Essential data used for determining the optimal composites for marine applications.

Laminate	TS-BSA (MPa)	TM-BSA (MPa)	FS-BSA (MPa)	FM-BSA (MPa)	TS-RR (%)	TM-RR (%)	FS-RR (%)	FM-RR (%)	SWAR (%)	TSR (%)	PFC (wt.%)	Density (g/cm^3^)	Thickness (mm)
GKKKG	82.26	5654.60	166.47	8539.36	79.73	77.97	73.72	82.53	4.30	3.40	19.19	1.244	3.455
GKFKF	88.98	6043.76	193.82	8635.09	86.77	78.57	88.36	93.56	3.79	3.47	18.61	1.267	3.277
GFKFG	103.38	6810.36	214.39	8926.69	79.82	75.83	71.40	97.69	4.20	3.53	21.59	1.284	2.597
GKGKG	96.46	6362.40	183.02	7477.83	70.23	75.68	72.47	84.64	4.03	3.37	14.81	1.298	2.861
Types of priorities	P	P	P	P	P	P	P	P	N	N	P	N	N

Note: P and N refer to positive and negative, respectively.

**Table 7:** Changes in priority rankings under sensitivity scenarios simulating a 25% increase in two main criteria and three sub-criteria.

Rank	Initial State	SEA	MC	TS-BSA	TM-BSA	FS-BSA
1	GKFKG (0.262)	GKFKG (0.243)	GFKFG (0.245)	GKFKG (0.243)	GKFKG (0.245)	GKFKG (0.245)
2	GFKFG (0.255)	GFKFG (0.234)	GKFKG (0.243)	GFKFG (0.243)	GFKFG (0.243)	GFKFG (0.244)
3	GKKKG (0.243)	GKGKG (0.227)	GKKKG (0.229)	GKGKG (0.229)	GKGKG (0.229)	GKGKG (0.226)
4	GKGKG (0.240)	GKKKG (0.226)	GKGKG (0.221)	GKKKG (0.226)	GKKKG (0.227)	GKKKG (0.226)

## Data Availability

The original contributions presented in this study are included in this article. Further inquiries can be directed to the corresponding authors.
